# Prevalence of post-traumatic stress symptoms among people influenced by coronavirus disease 2019 outbreak: A meta-analysis

**DOI:** 10.1192/j.eurpsy.2021.24

**Published:** 2021-04-12

**Authors:** Dan Qiu, Yilu Li, Ling Li, Jun He, Feiyun Ouyang, Shuiyuan Xiao

**Affiliations:** 1 Department of Social Medicine and Health Management, Xiangya School of Public Health, Central South University, Changsha, Hunan, China; 2 Mental Health Institute, Second Xian gya Hospital, Central South University, Changsha, Hunan, China

**Keywords:** COVID-19, meta-analysis, PTSD, prevalence

## Abstract

**Background:**

As one of the most widely researched consequence of traumatic events, the prevalence of post-traumatic stress symptoms among people exposed to the trauma resulting from coronavirus disease 2019 (COVID-19) outbreak varies greatly across studies. This review aimed at examining the pooled prevalence of post-traumatic stress symptoms among people exposed to the trauma resulting from COVID-19 outbreak.

**Methods:**

Systematic searches of databases were conducted for literature published on PubMed, EMBASE, Web of Science, the Cochrane Library, PsycArticle, and Chinese National Knowledge Infrastructure until October 14, 2020. Statistical analyses were performed using R software (PROSPERO registration number: CRD42020180309).

**Results:**

A total of 106,713 people exposed to the trauma resulting from the COVID-19 outbreak were identified in the 76 articles, of which 33,810 were reported with post-traumatic stress symptoms. The pooled prevalence of post-traumatic stress symptoms among people exposed to the trauma resulting from COVID-19 outbreak was 28.34%, with a 95% confidence interval of 23.03-34.32%. Subgroup analysis indicated that older age, male and bigger sample size were associated with higher prevalence of post-traumatic stress symptoms. After controlling for other factors, the results of meta-regression showed that the influence of gender and sample size on prevalence is no longer significant.

**Conclusions:**

Symptoms of post-traumatic stress disorder (PTSD) were very common among people exposed to the trauma resulting from COVID-19 outbreak. Further research is needed to explore more possible risk factors for post-traumatic stress symptoms and identify effective strategies for preventing PTSD-related symptoms among people exposed to the trauma resulting from COVID-19 outbreak.

## Background

As of December 14, 2020, 70.4 million confirmed cases of coronavirus disease 2019 (COVID-19) and 1.6 million deaths have been reported to the World Health Organization [1]. The outbreak of COVID-19 spread rapidly, caused enormous losses to individual health, national economy, and social wellbeing [2,3]. Currently, control of the epidemic of COVID-19 is still the dominant task across the world, millions of people are scared and even panic of the possible loss of health, life, and wealth. Although it is too early to predict how many people worldwide will be infected with this emerging virus, it is believed that the numbers of case and death will continue to increase in the forthcoming months.

Some psychologists draw attention toward post-traumatic stress disorder (PTSD) as the second tsunami of the COVID-19 pandemic [4]. According to the Diagnostic and Statistics of Mental Disorders, the fifth edition (DSM-5), the clinical features of PTSD include persistent avoidance of stimuli, persistent intrusion symptoms, negative alterations in cognition or mood and marked alterations in arousal and reactivity, all of which are related to traumatic events [5]. PTSD could cause clinically significant distress or impairment in occupational, social, or other important functioning [6]. The outbreak of COVID-19 is the most severe pandemic since Spanish Influenza, the outbreak itself and the measures taken to bring it under control have likely been highly stressful for many individuals, which is very likely to promote PTSD [7,8]. Additionally, it is said that such new type of infectious diseases were very traumatizing for people across the world with a poor understanding of viruses and spreading mechanisms [4]. The evocation of COVID-19 is thus generating a great anxiety and biased responses to threat, which can also promote PTSD [9].

When COVID-19 breaks out, people may experience many types of psychological trauma, such as directly suffering from the symptoms and traumatic treatment (respiratory failure, tracheotomy, etc.) [8], witness of suffering, struggling, and dying of patients [10]. Additionally, individuals may experience the fear of infection, social isolation, exclusion, and stigmatization, as patients, related caregivers, and workers, or even the general public [11,12]. It is said that there is a dose–response relationship between the degree of trauma and the mental health burden of disasters [13]. The prevalence of PTSD is higher among people who were directly exposed to the disaster, lower among related caregivers and rescue workers, and yet even lower in the general population. These different populations are likely to represent different levels of severity of trauma exposure, with direct victims having the highest exposure and associated PTSD prevalence while people in the general population having the lowest levels of exposure and associated PTSD prevalence [14]. Currently, the relationship between the degree of trauma and the mental health burden of COVID-19 outbreak is unclear.

As one of the most widely researched consequence of traumatic events [14], the prevalence of post-traumatic stress symptoms among people exposed to the trauma resulting from COVID-19 outbreak varies greatly across studies [15–18]. In addition, many factors have been reported to be associated with the prevalence of PTSD during COVID-19 outbreak, such as gender, age, and degree of trauma exposure [8,19,20], but the results are not consistent in different studies. The possible causes of the inconsistencies in the current estimates were unclear. For taking effective measures to reduce the psychological sequelae caused by COVID-19 across the world, it is necessary to determine a more accurate estimation of the prevalence of post-traumatic stress symptoms among people exposed to the trauma resulting from COVID-19 outbreak, and to explore the possible causes of the inconsistencies in the current estimates. This review aimed at examining the pooled prevalence of post-traumatic stress symptoms among people exposed to the trauma resulting from COVID-19 outbreak, summarizing possible vulnerability factors of post-traumatic stress symptoms and examining potentially vulnerable populations, try to provide a reference for COVID-19 and possible outbreak of infectious diseases in the future.

## Methods

This review was reported in accordance with the Preferred Reporting Items for Systematic Reviews and Meta-Analyses (PRISMA guideline) and Meta-analyses of Observational Studies in Epidemiology guidelines [21,22]. The protocol of this review is registered in the International Prospective Register of Systematic Reviews (registration number: CRD42020180309). See Supplementary Material for the details.

### Search strategy

PubMed, EMBASE, Web of Science, the Cochrane Library, MEDLINE, and Chinese National Knowledge Infrastructure were independently searched for published articles by two reviewers with no restrictions on date or language of publication up until 30 June 2020, and an update search was conducted on October 14, 2020. The following search terms were used: “COVID-19” (including “coronavirus disease 2019,” “SARS-CoV-2,” “severe acute respiratory syndrome coronavirus 2,” “COVID-19,” “Covid 19,” “SARS-CoV,” “novel coronavirus,” “coronavirus,” “CoV-2,” “2019-nCoV,” and “SARS COV2”); “Post-traumatic stress disorder” (including “post-traumatic stress disorder,” “post-traumatic syndrome,” “PTSD,” “stress disorder,” “post-traumatic,” and “post-traumatic syndrome”). See Supplementary Data for a full search strategy.

### Study selection

Studies were included if they meet the following criteria: (a) the study was observational study; (b) the participants were adult aged ≥18; (c) information about prevalence of post-traumatic stress symptoms among people exposed to the trauma resulting from COVID-19 outbreak was provided; (d) the full article was written in English or Chinese. Studies were excluded if (a) the report was a review, comments, meta-analysis, or protocol and (b) the participants with comorbid symptoms or chronic disease (such as mental illness, cancer, etc.).

### Data extraction

Data extraction was conducted independently in pairs by trained researchers who used standardized data extraction forms. Two reviewers (D.Q. and Y.L.L.) checked the titles, abstracts, and full-texts of the initial search results independently. Data were extracted on first author, country or area, survey period, target population, study design, sample size, response rate, percentage of male participants, mean age of participants, instruments used to identify post-traumatic stress symptoms, prevalence of post-traumatic stress symptoms, and quality score of the included studies. Any discrepancies that emerged in these procedures were discussed and resolved by involving a third reviewer (S.Y.X.).

### Quality assessment

Two independent reviewers (J.H. and F.Y.O.Y.) used the established guidelines, the Loney criteria, to evaluate the methodological quality of the included studies, which has been widely used to evaluate observational studies [23,24]. The included papers were scored according to eight criteria, such as study design, sample size, sampling method, response rate, definition of participants, appropriateness of measurement and analysis. The scores range from 0 to 8, with a score of 0–3 as low quality, 4–6 as moderate, and 7–8 as high [25]. See Table S3 for details on the quality assessment.

### Statistical analyses

When data were available for three or more papers, prevalence was combined [26]. When there were four or more papers, quantitative subgroup analysis was conducted [27]. All the statistical analyses were performed using the “meta” (4.12-0) and “metafor” package (2.4-0) of R version 4.0.0. Between-study heterogeneity was evaluated by Cochran’s Q test and quantified by the *I*^2^ statistic, with values >50% indicating moderate heterogeneity [28,29]. As we expected considerable heterogeneity, we calculated pooled prevalence with the random effects model. The pooled prevalence of post-traumatic stress symptoms among people exposed to the trauma resulting from COVID-19 outbreak was combined using Logit transformation method by a random effects model [29,30]. If more than one dataset was reported for the same group of participants, the outcomes that were assessed at the baseline were used. In order to compare the prevalence from different papers, we conducted subgroup meta-analysis. Because subgroup analyses should be interpreted with caution [31], we planned a priori to limit our subgroup analyses to a small number of baseline characteristics including gender, age, area, population, survey time after the outbreak, sample size, assessment tool, and quality score. The difference between subgroups was examined using the Cochran’s *Q* chi-square tests [30]. Mixed-model meta-regression analyses were performed by using Freeman–Tukey double arcsine method to explore potential moderators on the heterogeneity [32]. Publication bias was investigated by funnel plot and Egger’s test [30,33]. To evaluate the consistency of the results, sensitivity analysis was performed. In this study, sensitivity analyses were planned a priori for the primary analyses set by: (a) excluding studies one by one and (b) excluding studies with extreme outcomes [30,31]. All the statistical tests were two-sided, with a significance threshold of *p* < 0.05.

## Results

### Literature search

**Figure 1.**
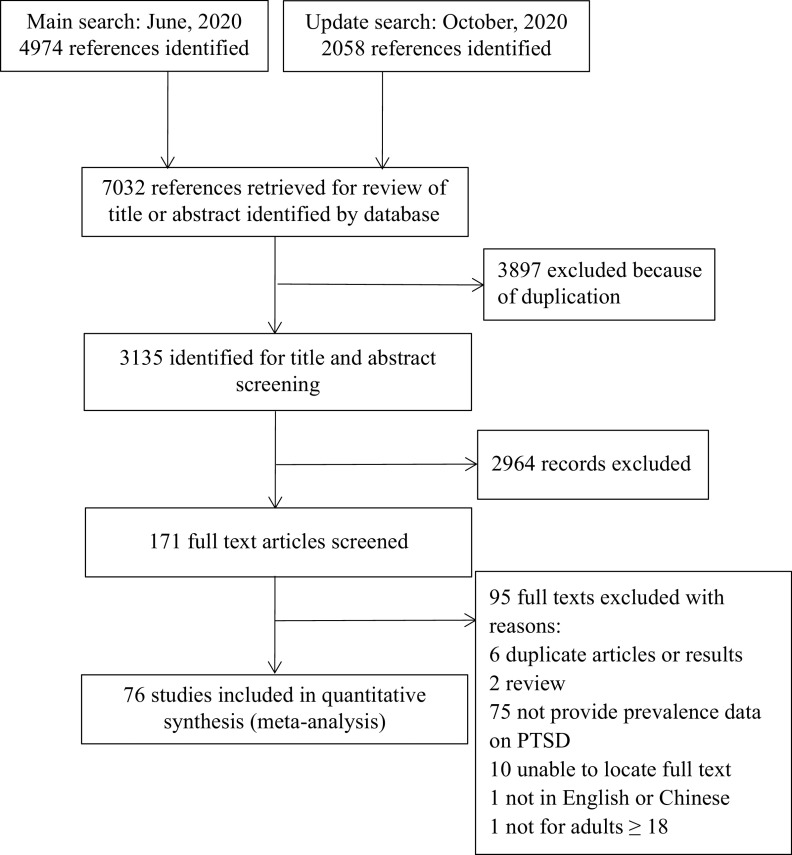
Flow of studies through review.

As shown in Figure 1, a total of 7,032 references were identified. Among them, 3,897 duplicates were removed. By screening titles and abstracts, 2,964 irrelevant articles were excluded. A total of 171 potentially relevant full-text articles were independently assessed based on the selection criteria. Further, 95 studies were excluded because of the following reasons: duplicate articles or results (*n* = 6); review or conference abstract (*n* = 2); did not provide data on PTSD (*n* = 75); unable to locate full text (*n* = 10); not in English or Chinese (*n* = 1); and not for participants aged ≥18 (*n* = 1). Finally, 76 eligible studies were included in this review [7–9,19,20,34–104]. The reliability for the full-text review between the two reviewers (D.Q. and Y.L.L.) was rated as good (Kappa = 0.78) [105]. See Figure 1 for the details.

### Study characteristics

Table 1 presents the main characteristics of the 76 included studies. Among them, 66 were in English and 10 were in Chinese. Most of the included studies were from Asia, such as China, Indian, and Singapore. See Table 1 for the details. From the 76 papers, 1 (1.31%) study was rated as high quality, 70 (92.11%) were rated as moderate, and 5 (6.58%) were rated as low quality. The reliability for the quality assessment between the two reviewers (J.H. and F.Y.O.Y.) was rated as good (Kappa = 0.73) [105]. For data extraction, all the criteria received a kappa value >0.85. Details of the methodological quality assessments of all 76 studies are showed in Tables S2 and S3.

**Table 1.** Study characteristics of the included studies.

First author	Study design	Population	Event/*N*	Survey time after the outbreak (week)	Mean age	Percentage of male participants (%)	Response rate (%)	Assessment tool	Quality score
Tee [34]	CS	General population	316/1,879	9	34.5 ± 13.4	31.0	75.4	IES-R (≥24)	5
Si [35]	CS	Healthcare workers	347/863	4	/	29.3	76.0	IES-6 (≥10)	6
Rodríguez-Rey [36]	CS	General population	1,559/3,055	7	32.1 ± 12.9	29.3	/	IES-R (≥24)	5
Nie [37]	CS	Healthcare workers	194/263	2	/	23.3	96.3	IES-R (≥20)	5
Liang [38]	CS	College students	1,822/4,164	3	/	52.0	/	IES-6	6
Li [39]	CC	Healthcare workers	1,382/4,369	2	/	0.0	82.2	IES-R (≥34)	7
Giusti [40]	CS	Healthcare workers	121/330	11	44.6 ± 13.5	37.4	71.2	IES-6 (≥9)	6
Chen [41]	CS	Healthcare workers/general population	900/1,493	4	/	55.3	93.3	IES-R (≥20)	6
Caillet [42]	F	General population	52/208	10	/	25.0	/	IES-R	5
Barbato [43]	CS	General population	33/148	10	41.4 ± 7.1	24.0	40.0	IES-R (≥33)	5
Alkhamees [44]	CS	General population	467/1,160	10	/	36.1	/	IES-R (≥24)	4
Zhou [45]	CC	General population	23/859	5	32.7	0.0	/	IES-R (≥33)	5
Zhao [46]	CS	General population	29/515	1	/	33.6	/	PCL-5	3
Zhang [47]	CS	General population	377/560	4	25.8 ± 2.7	0.0	93.3	IES-R (≥26)	4
Yin [48]	CS	Healthcare workers	15/371	2	35.3 ± 9.4	38.5	/	PCL-5 (≥33)	4
Wesemann [49]	CS	General population	23/60	6	59.0 ± 17.8	53.7	/	PCL-5	3
Wang [50]	F	General population	98/1,210	1	/	32.7	92.7	IES-R (≥24)	4
Varshney [51]	CS	General population	217/653	9	41.8	75.2	/	IES-R (≥24)	4
Traunmüller [52]	CS	General population	2,377/4,126	9	38.6 ± 13.3	26.0	/	IES-R (≥24)	5
Tang [53]	CS	General population	67/2,485	4	19.8	38.3	69.3	PCL-C (≥38)	6
Tan [54]	CS	General population	126/673	4	38.8 ± 7.4	74.4	50.8	IES-R (≥18)	5
Song [55]	CS	Healthcare workers	1,353/14,825	5	34.0 ± 8.2	35.7	/	PCL-C (≥38)	5
Sherman [56]	CS	General population	29/591	17	35.9 ± 8.2	22.5	35.3	PCL-5 (≥33)	6
Seyahi [57]	CS	Hospital workers/teachers	219/535,132/917	10	42.0/31.0/35.0	46.0/51.0/39.0	42.8/22.3/41.7	IES-R (≥33)	6
Rossi [58]	CS	General population	6,604/18,147	9	38.0 ± 23.0	20.5	/	GPS-PTSS	4
Rossi [59]	CS	Healthcare workers	681/1,379	9	39.0 ± 16.0	22.8	49.3	GPS-PTSD	6
Riello [60]	CS	Healthcare workers	433/1,071	15	/	24.6	53.0	IES-R (≥26)	6
Qi [61]	CS	COVID-19 patients	5/41	3	40.1 ± 10.1	41.9	52.4	PCL-5 (≥50)	5
Ma [62]	CS	General population	164/728	10	32.9 ± 10.4	29.8	72.8	IES-R (≥26)	6
Luceño-Moreno [63]	CS	Healthcare workers	160/1,422	9	43.8 ± 10.2	13.6	75.3	IES-R (≥20)	6
Liu [64]	CS	General population	20/285	1	/	45.6	95.0	PCL-5 (≥33)	4
Liu [20]	CS	COVID-19 patients	84/675	8	/	47.0	90.0	PCL-5	6
Liu [65]	CS	General population	285/898	11	24.5	14.1	/	PCL-C (≥38)	5
Li [66]	F	College students	160/1,442	2	/	/	71.2	IES-R (≥24)	7
Li [67]	CS	Healthcare workers	640/3,637	2	34.4 ± 9.6	37.0	/	IES-R (≥24)	3
Li [68]	CS	Healthcare workers	220/356	1	31.3	13.8	98.6	PCL-5	6
Li [69]	CS	General population	271/398	13	/	50.5	70.2	IES-7	5
Li [70]	CS	General population	744/1,109	9	/	56.0	/	IES-R (≥20)	5
Leng [71]	CS	Healthcare workers	5/90	7	/	27.8	83.3	PCL-C (≥50)	6
Le [72]	CS	General population	386/1,423	10	35.0	33.4	/	IES-R (≥24)	5
Lange [73]	CS	Healthcare workers	23/135	11	47.9 ± 11.4	40.9	31.1	IES-R	5
Lai [74]	CS	Healthcare workers	1,017/1,257	1	/	23.3	68.7	IES-R (≥26)	6
Lahav [8]	CS	General population	112/976	10	44.3 ± 14.2	18.4	77.3	PCL-5 (≥33)	5
Karatzias [75]	CS	General population	184/1,041	9	/	48.2	/	ITQ	6
Cardel [76]	CS	General population	92/250	10	/	15.0	/	IES-6	4
Guo [77]	CS	General population	1,944/2,441	1	/	47.6	/	PCL-C-2	5
González-Sanguino [78]	CS	General population	550/3,480	8	/	25.0	/	PCL-C	3
González Ramírez [79]	CS	General population	1,160/3,932	9	33.0	25.5	/	IES-R	4
Forte [80]	CS	General population	635/2,291	8	30.0 ± 11.5	25.4	/	IES-R(≥33)	5
Fekih-Romdhane [81]	CS	General population	199/603	10	29.2 ± 10.4	26.0	/	IES-R (≥33)	4
El‑Zoghby [82]	CS	General population	387/510	14	/	34.1	/	IES-R (≥24)	5
Dobson [83]	CS	Healthcare workers	93/320	12	/	18.4	/	IES-R (≥26)	6
Di Tella [84]	CS	Healthcare workers	38/145	8	42.9 ± 11.2	27.6	/	PCL-5	3
Cortés-Álvarez [85]	CS	General population	555/1,105	10	/	37.9	/	IES-R	6
Civantos [86]	CS	Healthcare workers	210/349	11	/	60.7	/	IES-R (≥26)	6
Civantos [87]	CS	Healthcare workers	43/163	15	/	74.2	23.3	IES-R (≥26)	5
Chi [88]	CS	College students	627/2,038	3	20.5 ± 1.9	37.0	81.5	PCL-C	5
Chew [89]	CS	Healthcare workers	91/1,146	14	31.7 ± 7.8	34.9	88.2	IES-R (≥24)	6
Chang [7]	CS	COVID-19 patients	13/64	6	54.7 ± 16.6	43.7	58.7	PCL-5 (≥33)	5
Cai [90]	CS	Healthcare workers	184/709	1	/	3.5	/	IES-R	5
Cai [19]	CS	COVID-19 patients	39/126	4	45.7 ± 14.0	47.6	100.0	PTSD-SS	4
Bo [9]	CS	COVID-19 patients	689/714	6	50.2 ± 12.9	49.1	97.8	PCL-C (⩾50)	5
Blekas [91]	CS	Healthcare workers	45/270	11	37.6 ± 11.9	21.9	/	PSDI-8	4
Zhang [92]	CS	General population	20/263	1	37.7 ± 14.0	40.3	65.7	IES-R	5
Zhang [93]	CS	Suspected COVID-19 patients	13/93	4	38.7 ± 13.6	54.8	100.0	PCL-5 (≥33)	6
Zhang [94]	CS	Suspected COVID-19 patients	87/306	6	34.8 ± 8.3	7.8	/	PCL-5 (≥38)	4
Yuan [95]	CS	Suspected COVID-19 patients	39/126	5	45.7 ± 14.0	47.6	/	PTSD-SS	4
Xie [96]	CS	General population	72/333	3	31.0 ± 10.1	39.9	93.8	PCL-C (≥40)	4
Liu [97]	CS	General population	453/584	3	35.3 ± 8.9	33.0	90.9	PCL-C (≥40)	6
Liu [98]	CS	Healthcare workers	20/221	8	/	1.0	99.0	PCL-C (≥40)	6
Leng [99]	CS	Healthcare workers	24/72	1	/	11.1	92.7	IES-R (≥26)	4
Chen [100]	CS	Healthcare workers	23/109	6	/	11.9	/	PCL-C (≥38)	6
Hao [101]	CC	General population	15/109	4	/	32.9/ 37.6	11.3/81.3	IES-R (≥24)	5
Liang [102]	CS	General population	84/584	2	/	38.1	95.7	PCL-C (≥38)	6
Li [103]	CS	Healthcare workers	104/205	3	/	14.6	99.9	PCL-C (≥38)	5
Huang [104]	CS	Healthcare workers	63/230	2	32.6 ± 6.2	18.7	93.5	PTSD-SS (≥55)	6

Abbreviations: CC, case–control study; COVID-19: coronavirus disease 2019; CS, cross-sectional study; F, follow up study; IES-6, The Impact of Event Scale-6; IES-R, The Impact of Event Scale-Revised; ITQ, The International Trauma Questionnaire; PCL-5, the Post‐traumatic stress disorder checklist‐5; PCL-C, The amended self-reported Post-Traumatic Stress Disorder (PTSD) Checklist-Civilian Version; PSDI-8, Posttraumatic Stress Disorder-8 inventory; PTSD-SS, Post-traumatic stress disorder self-rating scale.

### Pooled prevalence of post-traumatic stress symptoms among people influenced by the COVID-19 outbreak

**Figure 2.**
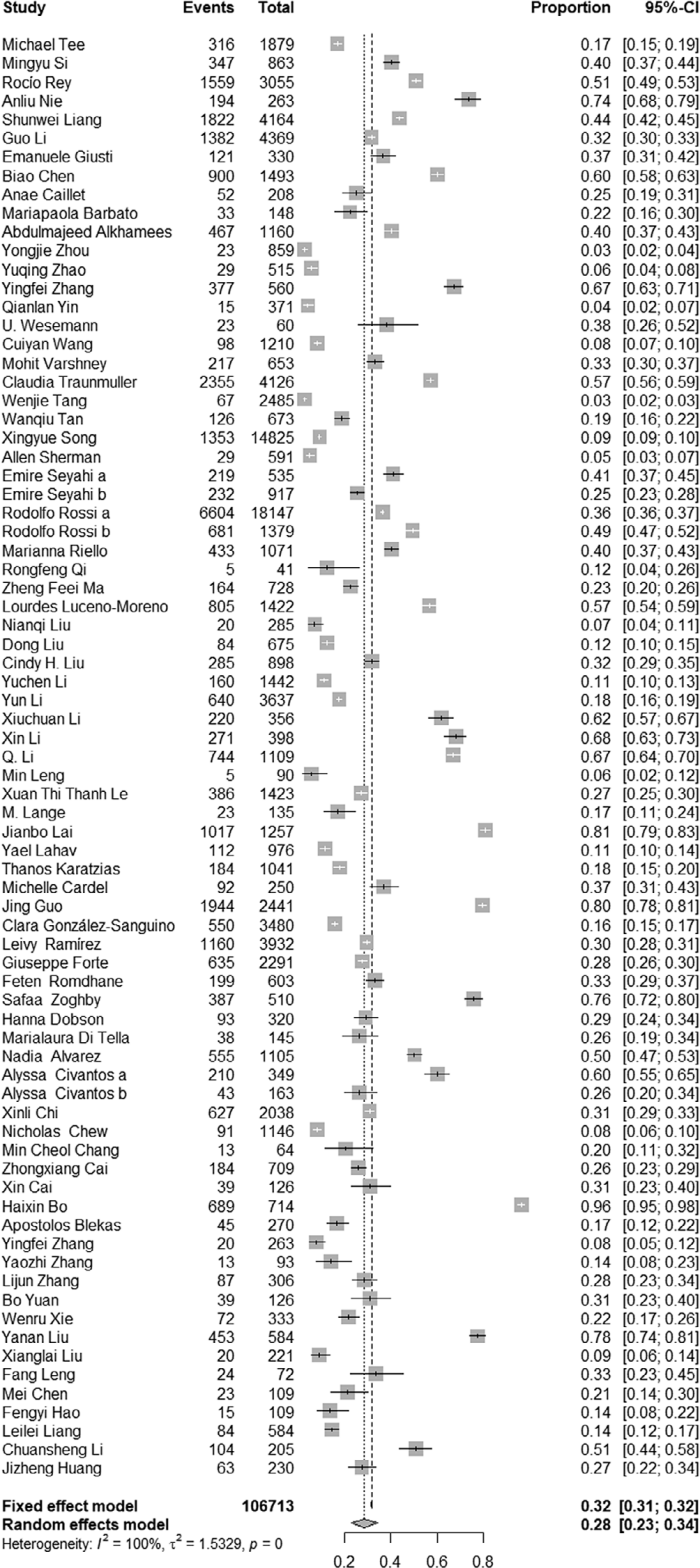
Forest plots.

There were 76 studies reported prevalence of post-traumatic stress symptoms among people exposed to the trauma resulting from the COVID-19 outbreak. The forest plot in Figure 2 depicts the details. A total of 106,713 people exposed to the trauma resulting from the COVID-19 outbreak were identified in the 76 articles, of which 33,810 were reported with post-traumatic stress symptoms. The random effects model was used to determine the pooled prevalence (*Q* = 14,854.51, *I*^2^ = 99.70%, *p* < 0.001), the pooled prevalence of post-traumatic stress symptoms among people exposed to the trauma resulting from COVID-19 outbreak was 28.34%, with a 95% confidence interval (CI) of 23.03–34.32%.

### Subgroup analysis for the included studies

The details of subgroup analyses are presented in Table 2. Significant differences in the prevalence of post-traumatic stress symptoms between different ages were found (*Q* = 221.97, *p* < 0.001). The results indicated that older participants (with a mean age ≥51) showed higher prevalence of post-traumatic stress symptoms (62.16%), younger participants (with a mean age between 18 and 20) showed lowest prevalence of post-traumatic stress symptoms (2.70%). Significant difference in the prevalence of post-traumatic stress symptoms between different gender was observed, the results indicated that studies with higher percentage of male participants (>50%) showed higher prevalence (26.70 vs. 41.79%; *Q* = 5.31, *p* = 0.021). The pooled prevalence of post-traumatic stress symptoms among people in the European region, the America region, the Eastern Mediterranean region, the Western Pacific region, and the South-East Asia region were 32.13%, 30.48%, 37.74%, 26.34%, and 17.16%, respectively. No significant differences in the prevalence of post-traumatic stress symptoms between different region were found (*Q* = 2.94, *p* = 0.580). Furthermore, the pooled prevalence of post-traumatic stress symptoms among people in the high-income region, the upper-middle-income region, and the lower-middle-income region were 30.03, 27.26, and 36.07%, respectively. No significant differences in the prevalence of post-traumatic stress symptoms between different income classification were found (*Q* = 0.81, *p* = 0.667). Also, the pooled prevalence of post-traumatic stress symptoms among COVID-19 patients, healthcare workers, suspected cases of COVID-19, the general population, and teachers/students were 36.30, 29.22, 24.47, 27.13, and 29.39%, respectively. No significant differences in the prevalence of post-traumatic stress symptoms between different population were found (*Q* = 0.87, *p* = 0.928). Although the prevalence of post-traumatic stress symptoms greater in earlier surveys (31.49%) than later surveys (25.79%), there were no significant differences in prevalence of post-traumatic stress symptoms between different survey time after the outbreak (*Q* = 1.05, *p* = 0.304). In addition, significant difference in the prevalence of post-traumatic stress symptoms between studies with different sample size was observed, articles with higher sample size showed lower prevalence (20.33 vs. 32.08%; *Q* = 6.61, *p* = 0.010). Studies used Impact of Event Scale (IES) as assessment tool showed higher prevalence (33.43%), studies used the amended self-reported PTSD Checklist—Civilian Version as assessment tool showed lowest prevalence (21.41%) and studies used Post-Traumatic Stress Disorder Self-Rating Scale, International Trauma Questionnaire, and Post-Traumatic Stress Disorder-8 Inventory showed moderate prevalence (28.96%). No significant differences in the prevalence of post-traumatic stress symptoms between studies used different assessment tools (*Q* = 3.47, *p* = 0.176). Lastly, no significant differences in the prevalence of post-traumatic stress symptoms between studies with different quality scores were observed (28.57 vs. 28.00%; *Q* = 0.01, *p* = 0.992).

**Table 2.** Subgroup analysis for the general population.

Subgroup	Studies	Pooled prevalence % (95% CI)	*I*^2^ (%)	Test of difference within each subgroup	
				*Q*	*p*
Mean age				221.97	<0.001
18–20	1	2.70 (2.13–3.41)	99.20		
21–30	5	37.58 (25.97–50.83)	98.90		
31–40	25	22.34 (15.74–31.15)	99.80		
41–50	10	26.53 (19.23–35.39)	96.50		
≥51	3	62.16 (13.84–94.38)	98.90		
Percentage of male participants (%)				5.31	0.021
0–50	65	26.70 (21.06–33.22)	99.70		
51–100	11	41.79 (30.64–53.86)	99.10		
WHO area				2.86	0.580
European	18	32.13 (26.04–38.89)	99.30		
Americas	8	30.48 (18.78–45.39)	99.20		
Eastern Mediterranean	4	37.74 (16.62–64.82)	99.40		
Western Pacific	45	26.34 (18.88–35.46)	99.70		
South-East Asia	2	17.16 (5.78–41.16)	98.80		
Income classification				0.81	0.667
High-income	26	30.03 (24.18–36.62)	99.40		
Upper-middle-income	45	27.26 (19.59–36.55)	99.70		
Lower-middle-income	5	36.07 (20.16–55.77)	99.30		
Population				0.87	0.928
COVID-19 patients	5	36.30 (8.86–76.96)	98.90		
Suspected COVID-19 patients	3	24.47 (17.00–33.89)	75.80		
Healthcare workers	26	29.22 (21.10–38.94)	99.50		
General population	39	27.13 (20.00–35.67)	99.80		
Teachers/students	4	29.39 (16.98–45.85)	99.40		
Survey time after outbreak (week)				1.05	0.304
1–4	35	31.49 (25.56–38.10)	99.50		
≥5	42	25.79 (18.17–35.23)	99.70		
Sample size				6.61	0.010
0–300	22	20.33 (15.86–25.68)	91.80		
≥301	55	32.08 (24.82–40.32)	99.80		
Diagnosis assessment				3.47	0.176
IER	42	33.43 (26.65–40.98)	99.60		
PCL-C	28	21.41 (13.44–32.34)	99.70		
Others	7	28.96 (21.62–37.59)	98.00		
Quality score				0.01	0.992
0–5	46	28.57 (21.80–36.45)	99.70		
≥6	31	28.00 (20.04–37.62)	99.70		

Abbreviations: COVID-19, coronavirus disease 2019; PCL-C, The amended self-reported Post-Traumatic Stress Disorder (PTSD) Checklist-Civilian Version; WHO, World Health Organization.

### Meta-regression analyses for the included studies

Table 3 presents the results of meta-regression analyses. Due to too much missing data (only 57.89% of studies reported data) on the mean age of participants, we were unable to include this variable in the meta-regression model. Bivariate meta-regression suggested that higher prevalence estimates reported in studies which used IES as assessment tool (*β* = −0.11, *p* = 0.061). Specifically, assessment tool accounted for 3.16% of the heterogeneity across studies, but the difference between different groups was not significant. In addition, area (*β* = −0.03, *p* = 0.568), income (*β* = 0.01, *p* = 0.882), population (*β* = 0.06, *p* = 0.626), percentage of male participants (*β* = 0.01, *p* = 0.473), survey time after the outbreak (*β* = 0.01, *p* = 0.775), quality score (*β* = 0.03, *p* = 0.407), and sample size (*β* = −0.01, p = 0.891) were not significant moderators too. Of the multivariate model, no significant moderators for heterogeneity were found (*p* > 0.05, *R*^2^ = 0.00%).

**Table 3.** Meta-regression analysis for the included studies.

Group	*Β*	95% CI		*p*	*R*^2^ (%)
		Lower	Upper		
Univariate analysis					
Area (Western Pacific vs. others)	−0.03	−0.14	0.08	0.568	0.00
Income (high income vs. others)	0.01	−0.11	0.13	0.882	0.00
Population (patients of COVID-19 vs. others)	0.06	−0.17	0.29	0.626	0.00
Percentage of male (continuous variable, %)	0.01	−0.01	0.01	0.473	0.00
Survey time after the outbreak (continuous variable)	0.01	−0.01	0.02	0.775	0.00
Quality score (continuous variable)	0.03	−0.03	0.08	0.407	0.00
Sample size (continuous variable)	−0.01	−0.01	0.01	0.891	0.00
Assessment tool (IES vs. others)	−0.11	−0.22	0.01	0.061	3.16
Multivariate analysis					0.00
Area (Western Pacific vs. others)	−0.11	−0.37	0.15	0.413	
Income (high income vs. others)	−0.02	−0.23	0.19	0.852	
Population (patients of COVID-19 vs. others)	0.15	−0.11	0.41	0.271	
Percentage of male (continuous variable, %)	0.01	−0.01	0.01	0.583	
Survey time after the outbreak (continuous variable)	−0.01	−0.03	0.01	0.372	
Quality score (continuous variable)	0.02	−0.04	0.09	0.388	
Sample size (continuous variable)	0.01	−0.01	0.01	0.879	
Assessment tool (IES vs. others)	−0.11	−0.24	0.01	0.069	

Abbreviations: COVID-19: coronavirus disease 2019; IES, The Impact of Event Scale.

### Publication bias and sensitivity analysis

**Figure 3.**
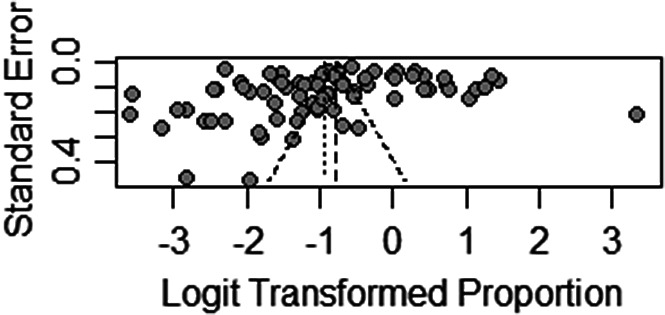
Funnel plot of publication bias.

Funnel plot of publication bias is presented in Figure 3. The funnel plot of publication bias is basically symmetric, but publication bias cannot be ruled out, so Egger’s test was conducted. The results of the Egger’s test showed that publication bias was not found in this study (*t* = −0.971, *p* = 0.334).

When each study was excluded one-by-one, the recalculated combined results did not change significantly. The pooled prevalence of post-traumatic stress symptoms ranged from 27.18% (95% CI: 22.40–32.55%) to 28.96% (95% CI: 23.72–34.81%), and the *I*^2^ statistic has remained at 99.7%. The results in the current study indicate that no individual study significantly influenced the overall results. In order to examine the influence of extreme cases on the current results, we excluded all the extreme results to conduct a sensitivity analysis (with a prevalence <5% or >70%). After removing eight studies [9,45,53,56,74,77,82,97], the pooled prevalence of post-traumatic stress symptoms was 26.20% (95% CI: 22.17–30.67%), and the *I*^2^ statistic was 99.5%. Compared with the initial results, no significant changes were found. See Figure S1 for the details of sensitivity analysis.

## Discussion

### Key findings

This review has highlighted the importance of considering the psychological impacts of people exposed to the trauma resulting from COVID-19 outbreak. A total of 106,713 people exposed to the trauma resulting from the COVID-19 outbreak were identified in the 76 articles, of which 33,810 were reported with post-traumatic stress symptoms. The pooled prevalence of post-traumatic stress symptoms among people exposed to the trauma resulting from COVID-19 outbreak was 28.34%, with a 95% CI of 23.03–34.32%.

### Comparison with the literature

Based on the results of the World Health Organization World Mental Health Surveys in 2017, the global lifetime prevalence of PTSD-related symptoms among the general population was 3.90–5.60% [106]. The pooled prevalence of post-traumatic stress symptoms among people exposed to the trauma resulting from COVID-19 outbreak in this study was 28.34%, which was much higher than the general population. Between December 2019 and May 2020, a few reviews related to mental health and infectious disease outbreak reported limited data on pooled prevalence of PTSD-related symptoms during the COVID-19 outbreak. In Salehi et al.’s [15] study, the pooled prevalence of PTSD-related symptoms during the coronavirus outbreaks (SARS, MERS, and COVID-19) was 18%, which was much lower than our results. In Krishnamoorthy et al.’s [16] study, the pooled prevalence of PTSD-related symptoms during the COVID-19 outbreak ranged from 21.94 to 27.00% [17,18], were lower than our results. Those researches have explored a variety of mental health problems (such as depression, anxiety, and insomnia), PTSD is only one of the outcomes. Thus, we believe their search strategies on PTSD are inaccurate enough and the data they included were limited, which may affect the results. Despite the high heterogeneity in their studies, no subgroup analysis was conducted to explore the source of the heterogeneity on the pooled prevalence of PTSD in most of them [15,16,18], which we think is another limitation. In Cooke et al.’s [17] study, although the source of the heterogeneity was explored, they only included age and gender as moderators. Moreover, we found that the pooled prevalence of post-traumatic stress symptoms among people exposed to the trauma resulting from COVID-19 outbreak was higher than flood survivors (15.74%) and hurricane survivors (17.81%) [107,108], but equal to earthquake survivors (23.66%) [109]. Compared with COVID-19 outbreak, some natural disasters such as flood and hurricane can be predicted, while earthquakes, infectious disease like COVID-19 were often happened suddenly and without a warning, pose a huge threat to people’s health and property in a short period of time [109]. Therefore, earthquakes and infectious disease outbreak might have caused more damage to people’s mental health than flood and hurricane.

The prevalence of post-traumatic stress symptoms in older adults is significantly higher than the younger people in the subgroup analysis, which was consistent with other studies [32]. Due to too much amount of missing data (only 57.89% of studies reported data on mean age of participants), we were unable to include this variable in the meta-regression model. Thus, this observation requires further clarification. Based on the current results, healthcare providers should pay more attention to the assessment of early trauma responses among older COVID-19 patients in the clinical practice and implement early psychological interventions accordingly. It is said that females were more likely to develop PTSD [109]. In the current study, however, males were associated with higher prevalence of post-traumatic stress symptoms. The possible reason is that males are more likely to be affected by COVID-19 and reported a higher fatality rate [110], they may experience higher level of severity of trauma exposure. In addition, although the epidemic situation is more serious in Europe and the Americas, no significant difference was found between different regions. It might be related to issues with numbers of studies, we think more prevalence studies in low-income countries are needed to understand the panorama of PTSD among people influenced by COVID-19. Besides, Previous research has shown that patients of infectious disease often directly suffering from the symptoms and traumatic treatment. After being cured, they were more vulnerable to social discrimination than other groups [14]. These experiences may result in higher prevalence of PTSD among them when compared with other populations. Although the prevalence of PTSD among COVID-19 patients is higher than that of other populations in this study, the difference is not significant, which need further exploration. Also, post-traumatic stress symptoms among people exposed to the trauma resulting from COVID-19 outbreak were higher in the immediate aftermath of the outbreak (0–4 weeks), but the difference was not significant, which was inconsistent with other studies [109,111,112]. The possible reason is that the epidemic has not abated over time and has been spreading, people have been exposed to the trauma resulting from COVID-19 outbreak. Thus, we think ongoing surveillance is essential. Furthermore, we found that the pooled prevalence of post-traumatic stress symptoms among people exposed to the trauma resulting from COVID-19 outbreak identified by different assessment tools was not significant. Studies have indicated that prevalence identified by screening tools were higher than prevalence identified by diagnostic tools [113]. However, all the included studies in this study were used screening tools, we were unable to explore the difference. It is note of worthy that some instruments derived from different conceptualization of the disorder and they may encompass different symptoms (e.g., Impact of Event Scale-Revised derived from the Diagnostic and Statistical Manual-Fourth Edition, Text Revision (DSM-IV-TR) while Post-Traumatic Stress Disorder Checklist-5 from DSM-5, including more symptoms than the former) [114]. Given that the included articles use many different screening tools, we think further research is needed to explore the influence of different screening tools on the prevalence of PTSD among people influenced by COVID-19 outbreak. It is reported that studies with poor methodological quality generally yielded more extreme prevalence estimates [115], the current study showed similar results. After controlling for other factors, however, the results of meta-regression showed that the influence of methodological quality on prevalence is no longer significant. Hence, this observation requires further clarification.

### Implications for the future

Epidemiological studies have demonstrated a rather high prevalence of mental health problems among different population after an epidemic of infectious disease [116–118]. While most of these mental health problems will fade out after the epidemic, symptoms of PTSD may last for a prolonged time and result in severe distress and disability [119]. In terms of applicability to COVID-19, we think ongoing surveillance is essential and healthcare policies need to take into account both short-term and long-term preventive strategy of PTSD. In addition, all the included studies identified PTSD by self-reporting questionnaires rather than clinical interviews by professional psychiatrists, as a consequence of which, the pooled prevalence of PTSD may have been overestimated. Only 6.39% reported data on COVID-19 patients. Thus, we think a large multicenter prospective study using a single validated measure of PTSD and measuring possible confounding factors in randomly selected COVID-19 patients is needed in the future, which would provide a more accurate estimate of PTSD among people influenced by COVID-19 outbreak, especially for COVID-19 patients. Although, there is little doubt that there is a dose–response relationship between the degree of trauma and the mental health burden of disasters [14], this relation may not necessarily mean that the principal mental health burden of people exposed to the trauma resulting from COVID-19 outbreak is among those who were most directly affected by the disease [13]. It will be important to establish whether indirect exposure to a trauma during a COVID-19 pandemic was correlated with higher risk of PTSD. Also, it is necessary to assess the relation between exposure to multiple traumas and risk of PTSD in the future. Additionally, subgroup analyses and the meta-regression analysis did not identify major sources of the heterogeneity although a high degree of heterogeneity between studies was observed. Therefore, there might be a considerable amount of uncertainty regarding the pooled prevalence of PTSD among people influenced by COVID-19 outbreak. Future research should, therefore, explore more potential risk factors for PTSD among people influenced by COVID-19 outbreak, especially genetic background as well as social support, previous traumatic events or concomitant psychiatric disorder [109,120].

### Limitations

Firstly, we excluded studies were not written in English or Chinese. Besides, although subgroup analyses and meta-regression analyses were conducted to control many moderating factors for the pooled prevalence of post-traumatic stress symptoms, heterogeneity was still remained in this review. It is reported that heterogeneity is difficult to avoid in meta-analysis of epidemiological surveys, [121] which suggesting the need for caution when drawing inferences about estimates of PTSD in post-disaster research. Additionally, although our study included relevant studies across 30 countries, more than half of the eligible studies were from upper-high income countries. Prevalence studies were scarce for many countries, especially for low-income countries. Considering the inconsistency of the healthcare environment and socioeconomic status across the world, more prevalence studies in low-income countries are needed to understand the panorama of PTSD among people influenced by COVID-19 outbreak. Also, we noticed that all the included studies were used screening tools to assess post-traumatic stress symptoms, no studies included were used diagnostic tools. It is possible that the pooled prevalence of post-traumatic stress symptoms caused by COVID-19 outbreak was overestimated in this review. Thus, we think ongoing surveillance is essential. Lastly, some included studies were investigated the prevalence before the time threshold from the first event (usually 30 days), we were unable to check this possible bias between studies. Although we explored the influence of survey time on the pooled prevalence, no significant result was found, which need further clarification.

## Conclusion

This review has highlighted the importance of considering the psychological impacts of people exposed to the trauma resulting from COVID-19 outbreak. A total of 106,713 people exposed to the trauma resulting from the COVID-19 outbreak were identified in the 76 articles, of which 33,810 were reported with post-traumatic stress symptoms. The pooled prevalence of post-traumatic stress symptoms among people exposed to the trauma resulting from COVID-19 outbreak was 28.34%, with a 95% CI of 23.03–34.32%. Further research is needed to explore more possible risk factors for post-traumatic stress symptoms and identify effective strategies for preventing and treating PTSD-related symptoms among people exposed to the trauma resulting from COVID-19 outbreak.

## Abbreviations

95% CI: 95% confidence interval;
COVID-19: coronavirus disease 2019;
DSM-5: diagnostic and statistics of mental disorders, the fifth edition;
IES-6: The Impact of Event Scale-6;
IES-R: The Impact of Event Scale-Revised;
ITQ: The International Trauma Questionnaire;
PCL-5: The Post-Traumatic Stress Disorder Checklist-5;
PCL-C: The amended self-reported Post-Traumatic Stress Disorder (PTSD) Checklist-Civilian Version;
PSDI-8: Post-Traumatic Stress Disorder-8 Inventory;
PTSD: post-traumatic stress disorder;
PTSD-SS: Post-Traumatic Stress Disorder Self-Rating Scale.

## Data Availability

The data that support the findings of this study are available in Table 1 and the Supplementary Material.

## Supplementary material

For supplementary material accompanying this paper visit http://dx.doi.org/10.1192/j.eurpsy.2021.24. click here to view supplementary material
