# Supplementary material for: Prevalence of post-traumatic stress symptoms among people influenced by coronavirus disease 2019 outbreak: A meta-analysis
Source: Eur Psychiatry. 2021 Apr 12;64(1):e30. doi: 10.1192/j.eurpsy.2021.24 (PMC8060540; doi:10.1192/j.eurpsy.2021.24)
Supplement: Supplementary file 1 [file S0924933821000249sup001.docx]

**Supplementary data**

**Search strategy**

**Table S1 Search strategy for PubMed:**

|  | **PTSD** |
| --- | --- |
| 1. | PTSD [Title/Abstract] |
| 2. | Stress Disorder [Title/Abstract] |
| 3. | Post Traumatic [Title/Abstract] |
| 4. | Posttraumatic [Title/Abstract] |
| 5. | Posttraumatic stress disorder [Title/Abstract] |
| 6. | Post-traumatic stress disorder* [Title/Abstract] |
| 7. | post traumatic syndrome* [Title/Abstract] |
| 8. | posttraumatic syndrome* [Title/Abstract] |
| 9 | 1 OR 2 OR 3 OR 4 OR 5 OR 6 OR 7 OR 8 |
| **b)** | **COVID-19** |
| 10. | COVID-19 [Title/Abstract] |
| 11. | Coronavirus disease 2019 [Title/Abstract] |
| 12. | Covid 19 [Title/Abstract] |
| 13. | severe acute respiratory syndrome coronavirus 2 [Title/Abstract] |
| 14. | SARS-CoV-2 [Title/Abstract] |
| 15. | SARS-CoV [Title/Abstract] |
| 16. | novel coronavirus [Title/Abstract] |
| 17. | coronavirus [Title/Abstract] |
| 18. | CoV-2 [Title/Abstract] |
| 19. | 2019-nCoV [Title/Abstract] |
| 20. | SARS COV2 [Title/Abstract] |
| 21. | 10 OR 11 OR 12 OR 13 OR 14 OR 15 OR 16 OR 17 OR 18 OR 19 OR 20 OR 20 |
| **c)** | **a) AND b)** |
| 23 | 9 AND 21 AND |

| Number | Item |
| --- | --- |
| A | Is the target population clearly defined? |
| B | Was either of the following ascertainment methods used [must be one or the other]? (1) probability sampling, or (2) entire population surveyed |
| C | Is the response rate >70% |
| D | Are non-responders clearly described? |
| E | Is the sample representative of the target population (>300 subjects)? |
| F | Were data collection methods standardized? |
| G | Were validated criteria used to assess for the presence/absence of disease? |
| H | Are the estimates of prevalence given with confidence intervals and in detail by subgroup (if applicable)? |

Table S2 The items of quality assessment

Table S3 The results of quality assessment

| study | A | B | C | D | E | F | G | H | Total score |
| --- | --- | --- | --- | --- | --- | --- | --- | --- | --- |
| Michael Tee (2020) | 0 | 0 | 1 | 0 | 1 | 1 | 1 | 1 | 5 |
| Mingyu Si (2020) | 1 | 1 | 1 | 0 | 1 | 1 | 1 | 0 | 6 |
| Rocío Rodríguez-Rey (2020) | 1 | 0 | 0 | 0 | 1 | 1 | 1 | 1 | 5 |
| Anliu Nie (2020) | 1 | 0 | 1 | 0 | 0 | 1 | 1 | 1 | 5 |
| Shunwei Liang (2020) | 1 | 1 | 0 | 0 | 1 | 1 | 1 | 1 | 6 |
| Guo Li (2020) | 1 | 1 | 1 | 0 | 1 | 1 | 1 | 1 | 7 |
| Emanuele Maria Giusti (2020) | 1 | 1 | 1 | 0 | 1 | 1 | 1 | 0 | 6 |
| Biao Chen (2020) | 1 | 0 | 1 | 0 | 1 | 1 | 1 | 1 | 6 |
| Anae¨ lle Caillet (2020) | 1 | 1 | 0 | 0 | 0 | 1 | 1 | 1 | 5 |
| Mariapaola Barbato (2020) | 1 | 1 | 0 | 0 | 0 | 1 | 1 | 1 | 5 |
| Abdulmajeed A. Alkhamees (2020) | 0 | 0 | 0 | 0 | 1 | 1 | 1 | 1 | 4 |
| Yongjie Zhou (2020) | 1 | 0 | 0 | 0 | 1 | 1 | 1 | 1 | 5 |
| Yuqing Zhao (2020) | 0 | 0 | 0 | 0 | 1 | 1 | 0 | 1 | 3 |
| Yingfei Zhang (2020) | 1 | 0 | 0 | 0 | 1 | 1 | 1 | 0 | 4 |
| Qianlan Yin (2020) | 1 | 0 | 0 | 0 | 1 | 1 | 1 | 0 | 4 |
| U. Wesemann (2020) | 1 | 0 | 0 | 0 | 0 | 1 | 0 | 1 | 3 |
| Cuiyan Wang (2020) | 0 | 0 | 1 | 0 | 1 | 1 | 1 | 0 | 4 |
| Mohit Varshney (2020) | 0 | 0 | 0 | 0 | 1 | 1 | 1 | 1 | 4 |
| Claudia Traunmüller (2020) | 1 | 0 | 0 | 0 | 1 | 1 | 1 | 1 | 5 |
| Wenjie Tang (2020) | 1 | 1 | 0 | 0 | 1 | 1 | 1 | 1 | 6 |
| Wanqiu Tan (2020) | 1 | 0 | 0 | 0 | 1 | 1 | 1 | 1 | 5 |
| Xingyue Song (2020) | 1 | 0 | 0 | 0 | 1 | 1 | 1 | 1 | 5 |
| Allen C. Sherman (2020) | 1 | 0 | 0 | 1 | 1 | 1 | 1 | 1 | 6 |
| Emire Seyahi (2020) | 1 | 1 | 0 | 0 | 1 | 1 | 1 | 1 | 6 |
| Rodolfo Rossi (2020) | 1 | 0 | 0 | 0 | 1 | 1 | 0 | 1 | 4 |
| Rodolfo Rossi (2020) | 1 | 1 | 0 | 0 | 1 | 1 | 1 | 1 | 6 |
| Marianna Riello (2020) | 1 | 1 | 0 | 0 | 1 | 1 | 1 | 1 | 6 |
| Rongfeng Qi (2020) | 1 | 1 | 0 | 0 | 0 | 1 | 1 | 1 | 5 |
| Zheng Feei Ma (2020) | 1 | 0 | 1 | 0 | 1 | 1 | 1 | 1 | 6 |
| Lourdes Luceño-Moreno (2020) | 1 | 0 | 1 | 0 | 1 | 1 | 1 | 1 | 6 |
| Nianqi Liu (2020) | 1 | 0 | 1 | 0 | 0 | 1 | 1 | 0 | 4 |
| Dong Liu (2020) | 1 | 1 | 1 | 0 | 1 | 1 | 0 | 1 | 6 |
| Cindy H. Liu (2020) | 1 | 0 | 0 | 0 | 1 | 1 | 1 | 1 | 5 |
| Yuchen Li (2020) | 1 | 1 | 1 | 0 | 1 | 1 | 1 | 1 | 7 |
| Yun Li (2020) | 0 | 0 | 0 | 0 | 1 | 1 | 1 | 0 | 3 |
| Xiuchuan Li (2020) | 1 | 1 | 1 | 0 | 1 | 1 | 0 | 1 | 6 |
| Xin Li (2020) | 1 | 0 | 0 | 0 | 1 | 1 | 1 | 1 | 5 |
| Q. Li (2020) | 1 | 0 | 0 | 0 | 1 | 1 | 1 | 1 | 5 |
| Min Leng (2020) | 1 | 1 | 1 | 0 | 0 | 1 | 1 | 1 | 6 |
| Xuan Thi Thanh Le (2020) | 1 | 0 | 0 | 0 | 1 | 1 | 1 | 1 | 5 |
| M. Lange (2020) | 1 | 1 | 0 | 0 | 0 | 1 | 1 | 1 | 5 |
| Jianbo Lai (2020) | 1 | 1 | 0 | 0 | 1 | 1 | 1 | 1 | 6 |
| Yael Lahav (2020) | 1 | 0 | 1 | 0 | 1 | 1 | 1 | 0 | 5 |
| Thanos Karatzias (2020) | 1 | 1 | 0 | 0 | 1 | 1 | 1 | 1 | 6 |
| Michelle I. Cardel (2020) | 1 | 0 | 0 | 0 | 0 | 1 | 1 | 1 | 4 |
| Jing Guo (2020) | 1 | 0 | 0 | 0 | 1 | 1 | 1 | 1 | 5 |
| Clara González-Sanguino (2020) | 0 | 0 | 0 | 0 | 1 | 1 | 1 | 0 | 3 |
| Leivy Patricia González Ramírez (2020) | 1 | 0 | 0 | 0 | 1 | 1 | 1 | 0 | 4 |
| Giuseppe Forte (2020) | 1 | 0 | 0 | 0 | 1 | 1 | 1 | 1 | 5 |
| Feten Fekih-Romdhane (2020) | 1 | 0 | 0 | 0 | 1 | 1 | 1 | 0 | 4 |
| Safaa M. El‑Zoghby (2020) | 1 | 0 | 0 | 0 | 1 | 1 | 1 | 1 | 5 |
| Hanna Dobson (2020) | 1 | 1 | 0 | 0 | 1 | 1 | 1 | 1 | 6 |
| Marialaura Di Tella (2020) | 1 | 0 | 0 | 0 | 0 | 1 | 0 | 1 | 3 |
| Nadia Yanet Cortés-Álvarez (2020) | 1 | 0 | 1 | 0 | 1 | 1 | 1 | 1 | 6 |
| Alyssa M. Civantos BA (2020) | 1 | 1 | 0 | 0 | 1 | 1 | 1 | 1 | 6 |
| Alyssa M. Civantos (2020) | 1 | 1 | 0 | 0 | 0 | 1 | 1 | 1 | 5 |
| Xinli Chi (2020) | 1 | 1 | 1 | 0 | 1 | 1 | 0 | 0 | 5 |
| Nicholas W. S. Chew (2020) | 1 | 0 | 1 | 0 | 1 | 1 | 1 | 1 | 6 |
| Min Cheol Chang (2020) | 1 | 1 | 0 | 0 | 0 | 1 | 1 | 1 | 5 |
| Zhongxiang Cai (2020) | 1 | 0 | 0 | 0 | 1 | 1 | 1 | 1 | 5 |
| Xin Cai (2020) | 1 | 1 | 1 | 0 | 0 | 1 | 0 | 0 | 4 |
| Haixin Bo (2020) | 1 | 0 | 0 | 0 | 1 | 1 | 1 | 1 | 5 |
| Apostolos Blekas (2020) | 1 | 0 | 0 | 0 | 0 | 1 | 1 | 1 | 4 |
| Yingfei Zhang (2020) | 1 | 1 | 0 | 0 | 0 | 1 | 1 | 1 | 5 |
| Yaozhi zhang (2020) | 1 | 1 | 1 | 0 | 0 | 1 | 1 | 1 | 6 |
| Lijun Zhang (2020) | 1 | 0 | 0 | 0 | 0 | 1 | 1 | 1 | 4 |
| Bo Yuan (2020) | 1 | 1 | 0 | 0 | 0 | 1 | 1 | 0 | 4 |
| Wenru Xie (2020) | 1 | 0 | 0 | 0 | 0 | 1 | 1 | 1 | 4 |
| Yanan Liu (2020) | 1 | 0 | 1 | 0 | 1 | 1 | 1 | 1 | 6 |
| Xianglai Liu (2020) | 1 | 1 | 1 | 0 | 0 | 1 | 1 | 1 | 6 |
| Fang Leng (2020) | 1 | 0 | 1 | 0 | 0 | 1 | 1 | 0 | 4 |
| Mei Chen (2020) | 1 | 1 | 1 | 0 | 0 | 1 | 1 | 1 | 6 |
| Fengyi Hao (2020) | 1 | 1 | 0 | 0 | 0 | 1 | 1 | 1 | 5 |
| Leilei Liang (2020) | 1 | 0 | 1 | 0 | 1 | 1 | 1 | 1 | 6 |
| Chuansheng Li (2020) | 1 | 0 | 1 | 0 | 0 | 1 | 1 | 1 | 5 |
| Jizheng Huang (2020) | 1 | 1 | 1 | 0 | 0 | 1 | 1 | 1 | 6 |


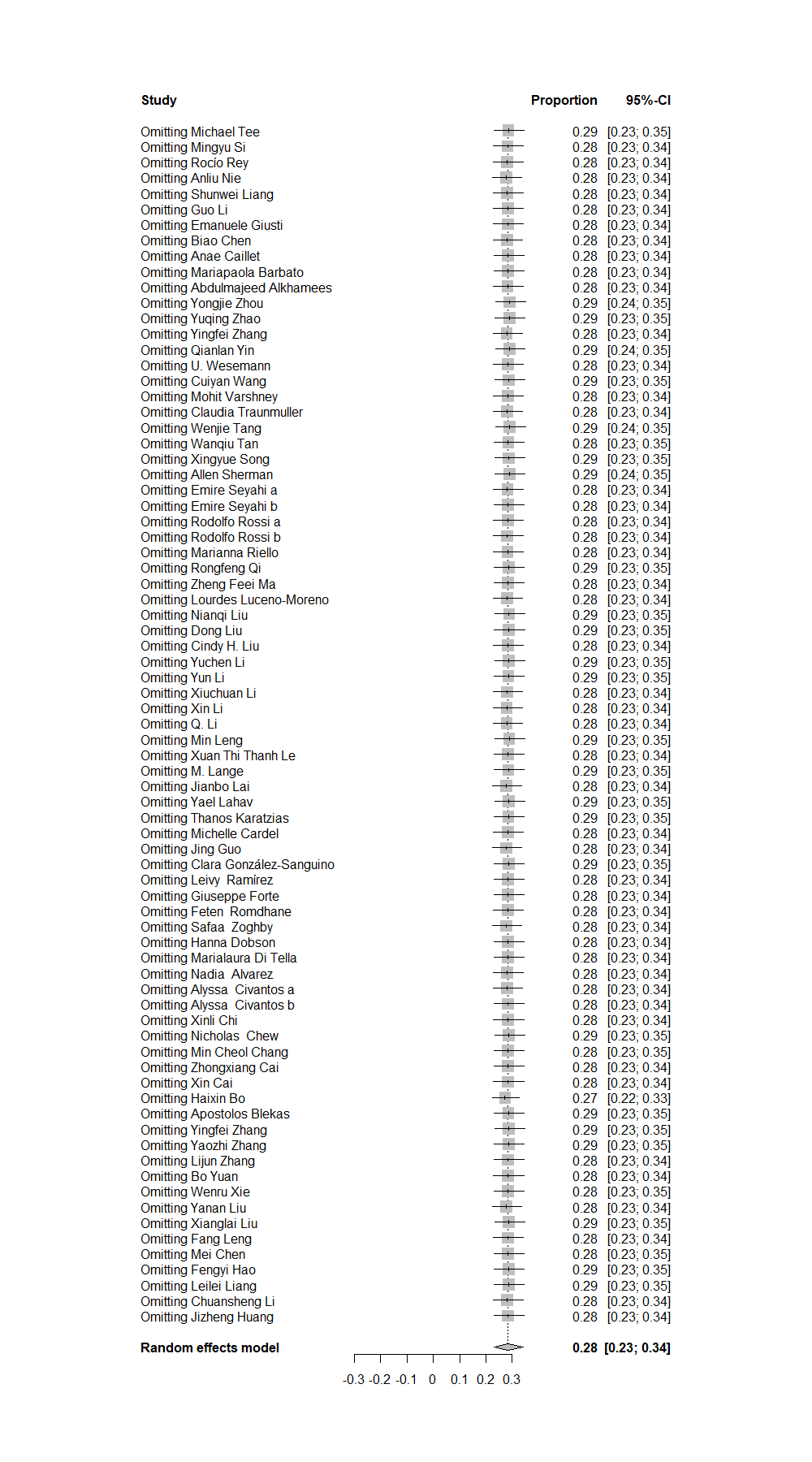


Fig. S1 Forest plots for the sensitivity analysis

**MOOSE Checklist**

**Prevalence of post-traumatic stress disorder among people influenced by COVID-19 outbreak: a systematic review and meta-analysis**

Dan Qiu, PhD

Department of Social Medicine and Health Management, Xiangya School of Public Health, Central South University

Yilu Li, PhD

Department of Social Medicine and Health Management, Xiangya School of Public Health, Central South University

Ling Li, PhD

Department of Social Medicine and Health Management, Xiangya School of Public Health, Central South University

Jun He, PhD

Department of Epidemiology and Health Statistics, Xiangya School of Public Health, Central South University

Feiyun Ouyang, PhD

Department of Epidemiology and Health Statistics, Xiangya School of Public Health, Central South University

Shuiyuan Xiao, MD

Department of Social Medicine and Health Management, Xiangya School of Public Health, Central South University;

Department of mental health, Xiangya Hospital, Central South University

Corresponding Author :

Shuiyuan Xiao

Address : Xiangya School of Public Health, Central South University, 110 Xiangya Road, Changsha, HUNAN, 410078, China.

E-mail : shuiyuanxiao1503@163.com

Phone : 86 731 84805454

Fax : 617-566-7805

| **Criteria** | | **Brief description of how the criteria were handled in the meta-analysis** |
| --- | --- | --- |
| **Reporting of background should include** | |  |
| √ | Problem definition | The psychological effects of COVID-19 outbreak can be deleterious and far-reaching. As one of the most widely researched consequence of traumatic events, the prevalence of PTSD symptoms among people exposed to the trauma resulting from COVID-19 outbreak varies greatly across studies. |
| √ | Hypothesis statement | The symptoms of PTSD are very common among people exposed to the trauma resulting from COVID-19 outbreak, relevant study characteristics, such as area, outcome measures, population have an impact on the outcome. |
| √ | Description of study outcomes | prevalence of PTSD symptoms |
| √ | Type of exposure or intervention used | people exposed to the trauma resulting from COVID-19 outbreak |
| √ | Type of study designs used | We included case-control studies, prospective cohort studies, cross-sectional studies |
| √ | Study population | people exposed to the trauma resulting from COVID-19 outbreak |
| **Reporting of search strategy should include** | |  |
| √ | Qualifications of searchers | The credentials of the two investigators DQ and YLL are indicated in the author list. |
| √ | Search strategy, including time period included in the synthesis and keywords | The following search terms were used: ‘COVID-19’ (coronavirus disease 2019, SARS-CoV-2, severe acute respiratory syndrome coronavirus 2 etc.); ‘Post-traumatic stress disorder’ (including Posttraumatic stress disorder, posttraumatic syndrome, PTSD, stress disorder, etc.). See supplementary data for a full search strategy.  We searched with no restrictions on date or language of publication up until 30 June 2020 and an update search was conducted on 14 October 2020. |
| √ | Databases and registries searched | PubMed, EMBASE, Web of Science, the Cochrane Library, PsycArticle, Chinese National Knowledge Infrastructure (CNKI), were independently searched by two reviewers, with no restrictions on date or language of publication up until 30 June 2020 and an update search was conducted on 14 October 2020. |
| √ | Search software used, name and version, including special features | We did not employ a search software. EndNote was used to merge retrieved citations and eliminate duplications |
| √ | Use of hand searching | We hand-searched bibliographies of retrieved papers for additional references |
| √ | List of citations located and those excluded, including justifications | Details of the literature search process are outlined in the flow chart and supplementary data. The citation list is available upon request |
| √ | Method of addressing articles published in languages other than English | We excluded studies not in English and Chinese |
| √ | Method of handling abstracts and unpublished studies | We planned to contacted authors for unpublished studies during the screening process when necessary, no such abstracts and unpublished studies appears in articles that meet the inclusion criteria at last. |
| √ | Description of any contact with authors | Not applicable (All articles that meet the inclusion criteria have complete data for pooled prevalence) |
| **Reporting of methods should include** | |  |
| √ | Description of relevance or appropriateness of studies assembled for assessing the hypothesis to be tested | Detailed inclusion and exclusion criteria were described in the methods section. |
| √ | Rationale for the selection and coding of data | Two reviewers (DQ and YLL) checked the titles, abstracts and full-texts of the initial search results independently. Data were extracted on first author, year of publication, country or area, survey period, sample size, response rate, percentage of male participants, average age of participants, instruments used to identify PTSD symptoms, prevalence of PTSD symptoms, quality score of the included studies, etc. Any discrepancies that emerged in these procedures were discussed and resolved by involving a third reviewer (SYX). |
| √ | Assessment of confounding | In order to compare the prevalence from different studies (such as survey time after the outbreak, type of disease, diagnostic method, population etc.), we conducted subgroup meta-analysis. The difference between subgroups was examined using the Cochran's Q chi-square tests. Mixed-model meta-regression analyses were performed by using Freeman-Tukey double arcsine method to explore potential moderators on the heterogeneity. |
| √ | Assessment of study quality, including blinding of quality assessors; stratification or regression on possible predictors of study results | Two independent reviewers (JH and FYOY) used the established guidelines, the Loney criteria, to evaluate the methodological quality of the included studies, which has been widely used to evaluate observational studies. The included papers were scored according to eight criteria, such as definition of participants, study design, sampling method, response rate, sample size, appropriateness of measurement and analysis. The scores range from 0 to 8, with a score of 0-3 as low quality, 4-6 as moderate and 7-8 as high. See Table S3 for details on the quality assessment.  Publication bias was investigated by funnel plot and Egger's test. |
| √ | Assessment of heterogeneity | Heterogeneity of the studies were explored within two types of study designs using Cochrane’s Q test of heterogeneity and I^2^ statistic that provides the relative amount of variance of the summary effect due to the between-study heterogeneity. |
| √ | Description of statistical methods in sufficient detail to be replicated | Description of methods of meta-analyses, sensitivity analyses, meta-regression and assessment of publication bias are detailed in the methods. |
| √ | Provision of appropriate tables and graphics | We included 1 flow chart,1 summary table, 1 forest plot of all studies, 1 funnel plot of publication bias, 1 table of subgroup analyses and 1 table of meta-regression analysis.  In addition, we included 1 supplementary Figs and 3 supplementary tables in the supplementary data file. |
| **Reporting of results should include** | |  |
| √ | Graph summarizing individual study estimates and overall estimate | Figure 2 |
| √ | Table giving descriptive information for each study included | Table 1 |
| √ | Results of sensitivity testing | Fig S1 |
| √ | Indication of statistical uncertainty of findings | 95% confidence intervals were presented with all summary estimates, I^2^ values and results of sensitivity analyses |
| **Reporting of discussion should include** | |  |
| √ | Quantitative assessment of bias | The results of the Egger's test showed that publication bias was not found in this study and the sensitivity analysis showed that no individual study significantly influenced the overall results. However, the observed heterogeneity should be noticed. |
| √ | Justification for exclusion | We excluded studies that not write in English or Chinese, which was a limitation in this review. |
| √ | Assessment of quality of included studies | We discussed the results of the subgroup analyses, and potential reasons for the observed heterogeneity. |
| **Reporting of conclusions should include** | |  |
| √ | Consideration of alternative explanations for observed results | We noted that the variations in the prevalence may be due to true population differences, or to differences in quality of studies, survey time, etc. |
| √ | Generalization of the conclusions | Evidence suggests that the symptoms of PTSD were very common among people exposed to the trauma resulting from COVID-19 outbreak and may last for a prolonged time. Healthcare policies need to take into account both short-term and long-term preventive strategy of PTSD in the forthcoming months. |
| √ | Guidelines for future research | At first, healthcare policies need to take into account both short-term and long-term preventive strategy of PTSD in the forthcoming months. It will be important to establish whether indirect exposure to a trauma during a COVID-19 pandemic was correlated with higher risk of PTSD. Also, it is necessary to assess the relation between exposure to multiple traumas and risk of PTSD in the future. Additionally, we think a large multicenter prospective study using a single validated measure of PTSD and measuring possible confounding factors in randomly selected participants is needed in the future, which would provide a more accurate estimate of PTSD among survivors of COVID-19. At last, we think ongoing surveillance is essential. |
| √ | Disclosure of funding source | This research was supported by the Ministry of Science and Technology of China (Grant NO: 2016YFC0900802). The funding agency did not take part in the design of the study and collection, analysis, and interpretation of data and in writing the manuscript. |

**PRISMA checklist**

| **Section / topic** | **#** | **Checklist item** | **Reported on page #** |
| --- | --- | --- | --- |
| **TITLE** | | |  |
| Title | 1 | Prevalence of post-traumatic stress disorder among people influenced by COVID-19 outbreak: a meta-analysis | Title |
| **ABSTRACT** | | |  |
| Structured summary | 2 | Background: The psychological effects of COVID-19 outbreak can be deleterious and far-reaching. As one of the most widely researched consequence of traumatic events, the prevalence of PTSD symptoms among people exposed to the trauma resulting from COVID-19 outbreak varies greatly across studies.  Aims: This review aimed at examining the pooled prevalence of PTSD symptoms among people exposed to the trauma resulting from COVID-19 outbreak, summarizing possible vulnerability factors of PTSD symptoms and examining potentially vulnerable populations  Method: Systematic searches of databases were conducted for literature published on PubMed, EMBASE, Web of Science, the Cochrane Library, PsycArticle, CNKI until 14 October 2020. Statistical analyses were performed using R software (PROSPERO registration number: CRD42020182366).  Results: A total of 106713 people exposed to the trauma resulting from the COVID-19 outbreak were identified in the 76 articles, of which 33810 were reported with PTSD symptoms. The random effects model was used to determine the pooled prevalence (Q= 14854.51, I2 = 99.70%, P < 0.001), the pooled prevalence of PTSD symptoms among people exposed to the trauma resulting from COVID-19 outbreak was 28.34%, with a 95% CI of 23.03% to 34.32%. Factors including male, bigger sample size were associated with higher prevalence of PTSD symptoms. After controlling for other factors, the results of meta-regression showed that the influence of gender and sample size on prevalence is no longer significant.  Conclusions: Evidence suggests that the symptoms of PTSD were very common among people exposed to the trauma resulting from COVID-19 outbreak. Further research is needed to explore more possible risk factors for PTSD and identify effective strategies for preventing and treating PTSD symptoms among people exposed to the trauma resulting from COVID-19 outbreak. | Abstract |
| **INTRODUCTION** | | |  |
| Rationale | 3 | As of December 14, 2020, 70.4 million confirmed cases of COVID-19 and 1.6 million deaths have been reported to the World Health Organization (WHO). The outbreak of COVID-19 spread rapidly, caused enormous losses to individual health, national economy, and social wellbeing. Currently, control of the epidemic of COVID-19 is still the dominant task across the world, millions of people are scared and even panic of the possible loss of health, life and wealth. Although it is too early to predict how many people worldwide will be infected with this emerging virus, it is believed that the numbers of case and death will continue to increase in the forthcoming months. As one of the most widely researched consequence of traumatic events, the prevalence of PTSD symptoms and risk factors among people exposed to the trauma resulting from COVID-19 outbreak varies greatly across studies. For taking effective measures to reduce the psychological sequelae caused by COVID-19 outbreak across the world, determine a more accurate estimation of the prevalence of PTSD symptoms, understanding how COVID-19 outbreak cause PTSD and who might be vulnerable are essential. | Introduction |
| Objectives | 4 | This review aimed at examining the pooled prevalence of PTSD symptoms among people exposed to the trauma resulting from COVID-19 outbreak, summarizing possible vulnerability factors of PTSD symptoms and examining potentially vulnerable populations, try to provide a reference for COVID-19 and possible outbreak of infectious diseases in the future. | Introduction |
| **METHODS** | | |  |
| Protocol and registration | 5 | This review was reported in accordance with the PRISMA guideline and MOOSE guidelines. The protocol of this review is registered in the International Prospective Register of Systematic Reviews (registration number: CRD42020180309). | Methods |
| Eligibility criteria | 6 | Studies were included if they meet the following criteria: (1) the study was observational study; (2) the participants were adult aged ≥18; (3) information about prevalence of PTSD symptoms among people exposed to the trauma resulting from COVID-19 outbreak; (4) the full article was written in English or Chinese. Studies were excluded if (1) the report was a review, comments, meta-analysis or protocol; (2) the participants with comorbid symptoms or had a chronic disease. | Methods |
| Information sources | 7 | PubMed, EMBASE, Web of Science, the Cochrane Library, MEDLINE, Chinese National Knowledge Infrastructure (CNKI), were independently searched by two reviewers, with no restrictions on date or language of publication up until 30 June 2020 and an update search was conducted on 14 October 2020. The following search terms were used: ‘COVID-19’ (coronavirus disease 2019, SARS-CoV-2, severe acute respiratory syndrome coronavirus 2 etc.); ‘Post-traumatic stress disorder’ (including Posttraumatic stress disorder, posttraumatic syndrome, PTSD, stress disorder, etc.). See supplementary data for a full search strategy. | Methods |
| Search | 8 | 1. **PTSD** 2. PTSD **[Title/Abstract]** 3. Stress Disorder **[Title/Abstract]** 4. Post Traumatic **[Title/Abstract]** 5. Posttraumatic **[Title/Abstract]** 6. Posttraumatic stress disorder **[Title/Abstract]** 7. Post-traumatic stress disorder* **[Title/Abstract]** 8. post traumatic syndrome* **[Title/Abstract]** 9. posttraumatic syndrome* **[Title/Abstract]** 10. 1 OR 2 OR 3 OR 4 OR 5 OR 6 OR 7 OR 8 11. **COVID-19** 12. COVID-19 **[Title/Abstract]** 13. Coronavirus disease 2019 **[Title/Abstract]** 14. Covid 19 **[Title/Abstract]** 15. severe acute respiratory syndrome coronavirus 2 **[Title/Abstract]** 16. SARS-CoV-2 **[Title/Abstract]** 17. SARS-CoV **[Title/Abstract]** 18. novel coronavirus **[Title/Abstract]** 19. coronavirus **[Title/Abstract]** 20. CoV-2 **[Title/Abstract]** 21. 2019-nCoV **[Title/Abstract]** 22. SARS COV2 **[Title/Abstract]** 23. 10 OR 11 OR 12 OR 13 OR 14 OR 15 OR 16 OR 17 OR 18 OR 19 OR 20 24. 9 AND 21 | Supplementary data |
| Study selection | 9 | Two reviewers (DQ and YLL) checked the titles, abstracts and full-texts of the initial search results independently. Data were extracted on first author, year of publication, country or area, survey period, sample size, response rate, percentage of male participants, average age of participants, instruments used to identify PTSD symptoms, prevalence of PTSD symptoms, quality score of the included studies, etc. Any discrepancies that emerged in these procedures were discussed and resolved by involving a third reviewer (SYX). | Methods |
| Data collection process | 10 | Two reviewers (DQ and YLL) checked the titles, abstracts and full-texts of the initial search results independently. Any discrepancies that emerged in these procedures were discussed and resolved by involving a third reviewer (SYX). | Methods |
| Data items | 11 | Data were extracted on first author, year of publication, country or area, survey period, sample size, response rate, percentage of male participants, average age of participants, instruments used to identify PTSD symptoms, prevalence of PTSD symptoms, quality score of the included studies, etc. | Methods |
| Risk of bias in individual studies | 12 | Two independent reviewers (JH and FYOY) used the established guidelines, the Loney criteria, to evaluate the methodological quality of the included studies, which has been widely used to evaluate observational studies. The included papers were scored according to eight criteria, such as definition of participants, study design, sampling method, response rate, sample size, appropriateness of measurement and analysis. The scores range from 0 to 8, with a score of 0-3 as low quality, 4-6 as moderate and 7-8 as high. See Table S3 for details on the quality assessment. | Methods |
| Summary measures | 13 | prevalence of PTSD symptoms | Methods |
| Synthesis of results | 14 | When data were available for three or more studies, prevalence or risk factor was combined. When there were 10 or more studies, quantitative subgroup analysis was conducted. All the statistical analyses were performed using the “meta” (4.12-0) and “metafor” package (2.4-0) of R version 4.0.0. Between-study heterogeneity was evaluated by Cochran's Q test and quantified by the I^2^ statistic, with values 50% or more indicating possible heterogeneity. The pooled prevalence of PTSD symptoms was combined using Logit transformation method by a random effects model if significant heterogeneity was observed across studies (when P < 0.05, I^2^ > 50%). If more than one dataset was reported for the same group of participants, the outcomes that were assessed at the baseline were used. | Methods |

Page 1 of 2

| **Section/topic** | **#** | **Checklist item** | **Reported on page #** |
| --- | --- | --- | --- |
| Risk of bias across studies | 15 | Publication bias was investigated by funnel plot and Egger's test. To evaluate the consistency of the results, sensitivity analysis was performed by removing each study individually. All the statistical tests were 2-sided, with a significance threshold of P < 0.05. | Methods |
| Additional analyses | 16 | In order to compare the prevalence from different studies (such as survey time after the outbreak, area, diagnostic method, population etc.), we conducted subgroup meta-analysis. The difference between subgroups was examined using the Cochran's Q chi-square tests. Mixed-model meta-regression analyses were performed by using Freeman-Tukey double arcsine method to explore potential moderators on the heterogeneity. | Methods |
| **RESULTS** | | |  |
| Study selection | 17 | As shown in Fig. 1, a total of 7032 references were identified. Among them, 3897 duplicates were removed. By screening titles and abstracts, 3139 irrelevant articles were excluded. A total of 171 potentially relevant full-text articles were independently assessed based on the selection criteria. Further, 95 studies were excluded because of the following reasons: duplicate articles or results (n = 6); review or conference abstract (n = 2); did not provide data on PTSD (n = 75); unable to locate full text (n = 10); not in English or Chinese (n = 1); not for participants aged ≥18 (n=1). Finally, 76 eligible studies were included in this review. See Fig. 1 for the details. | Results |
| Study characteristics | 18 | Table 1 presents the main characteristics of the 76 included studies. Among them, 66 were in English and 10 were in Chinese. Most of the included studies were from Asia, such as China, Indian and Singapore. See Table 1 for the details. From the 76 papers, one (1.31%) studies were rated as high quality, 70 (92.11%) were rated as moderate, and five (6.58%) were rated as low quality. Details of the methodological quality assessments of all 77 studies are showed in Additional File 2. | Results |
| Risk of bias within studies | 19 | From the 76 papers, one (1.31%) studies were rated as high quality, 70 (92.11%) were rated as moderate, and five (6.58%) were rated as low quality. Details of the methodological quality assessments of all 77 studies are showed in Additional File 2. | Results |
| Results of individual studies | 20 | There were 77 studies reported prevalence of PTSD symptoms among people exposed to the trauma resulting from the COVID-19 outbreak. The forest plot in Fig. 2 depicts the details. A total of 107738 people exposed to the trauma resulting from the COVID-19 outbreak were identified in the 77 articles, of which 34032 were reported with PTSD symptoms. The random effects model was used to determine the pooled prevalence (I2 = 99.70%, P < 0.001), the pooled prevalence of PTSD symptoms among people exposed to the trauma resulting from COVID-19 outbreak was 28.24%, with a 95% CI of 23.01% to 34.14%. | Results |
| Synthesis of results | 21 | There were 76 studies reported prevalence of PTSD symptoms among people exposed to the trauma resulting from the COVID-19 outbreak. The forest plot in Fig. 2 depicts the details. A total of 106713 people exposed to the trauma resulting from the COVID-19 outbreak were identified in the 76 articles, of which 33810 were reported with PTSD symptoms. The random effects model was used to determine the pooled prevalence (Q= 14854.51, I2 = 99.70%, P < 0.001), the pooled prevalence of PTSD symptoms among people exposed to the trauma resulting from COVID-19 outbreak was 28.34%, with a 95% CI of 23.03% to 34.32%. | Results |
| Risk of bias across studies | 22 | Funnel plot of publication bias is presented in Fig. 3. The funnel plot of publication bias is basically symmetric, but publication bias cannot be ruled out, so Egger's test was conducted. The results of the Egger's test showed that publication bias was not found in this study (t = -0.971, p = 0.334). When each study was excluded one-by-one, the recalculated combined results did not change significantly. The pooled prevalence of PTSD symptoms ranged from 27.18% (95% CI: 22.40%-32.55%) to 28.96% (95% CI: 23.72%-34.81%), and the I2 statistic has remained at 99.7%. The results in the current study indicate that no individual study significantly influenced the overall results. | Results |
| Additional analysis | 23 | The details of subgroup analyses are presented in Table 2. Significant differences in the prevalence of PTSD symptoms between different age was found (Q=221.97, P < 0.001). The results indicated that older participants (with a mean age ≥ 51) showed higher prevalence of PTSD (62.16%), younger participants (with a mean age between 18 and 20) showed lowest prevalence of PTSD (2.70%). Significant difference in the prevalence of PTSD symptoms between different gender was observed, the results indicated that studies with higher percentage of male participants (> 50%) showed higher prevalence (26.70% vs. 41.79%; Q= 5.31, P = 0.021). The pooled prevalence of PTSD symptoms among people in the European region, the America region, the Eastern Mediterranean region, the Western Pacific region and the South-East Asia region was 32.13%, 30.48%, 37.74%, 26.34% and 17.16%, respectively. No significant differences in the prevalence of PTSD symptoms between different region was found (Q= 2.94, P = 0.580). Furthermore, the pooled prevalence of PTSD symptoms among people in the high-income region, the upper-middle-income region, and the lower-middle-income region was 30.03%, 27.26% and 36.07%, respectively. No significant differences in the prevalence of PTSD symptoms between different income classification was found (Q= 0.81, P = 0.667). Also, the pooled prevalence of PTSD symptoms among COVID-19 patient, healthcare workers, suspected cases of COVID-19, the general population and teachers/students was 36.30%, 29.22%, 24.47%, 27.13% and 29.39%, respectively. No significant differences in the prevalence of PTSD symptoms between different population was found (Q= 0.87, P = 0.928). Although the prevalence of PTSD symptoms greater in earlier surveys (31.49%) than later surveys (25.79%), there were no significant differences in prevalence of PTSD symptoms between different survey time after the outbreak (Q= 1.05, P = 0.304). In addition, significant difference in the prevalence of PTSD symptoms between studies with different sample size was observed, articles with higher sample size showed lower prevalence (20.33% vs. 32.08%; Q= 6.61, P = 0.010). Studies used IES as assessment tool showed higher prevalence (33.43%) and Studies used PCL-C as assessment tool showed lowest prevalence (21.41%). No significant differences in the prevalence of PTSD symptoms between studies used different assessment tools (33.43% vs. 21.41% vs. 28.96%; Q= 3.47, P = 0.176). Lastly, no significant differences in the prevalence of PTSD symptoms between studies with different quality scores was observed (28.57% vs. 28.00%; Q= 0.01, P = 0.992).  Table 3 presents the results of meta-regression analyses. Bivariate meta-regression suggested that higher prevalence estimates reported in studies which used IES as assessment tool (β = −0.11, p = 0.061). Specifically, assessment tool accounted for 3.16% of the heterogeneity across studies, but the difference between different groups was not significant. In addition, area (β = −0.03, p = 0.568), income (β = 0.01, p = 0.882), population (β = 0.06, p = 0.626), percentage of male participants (β = 0.01, p = 0.473), survey time after the outbreak (β = 0.01, p = 0.775), quality score (β = 0.03, p = 0.407) and sample size (β = −0.01, p = 0.891) were not significant moderators too. Of the multivariate model, no significant moderators for heterogeneity were found (P > 0.05, R2=0.00%). | Results |
| **DISCUSSION** | | |  |
| Summary of evidence | 24 | This review has highlighted the importance of considering the psychological impacts of people exposed to the trauma resulting from COVID-19 outbreak. A total of 106713 people exposed to the trauma resulting from the COVID-19 outbreak were identified in the 76 articles, of which 33810 were reported with PTSD symptoms. The pooled prevalence of PTSD symptoms among people exposed to the trauma resulting from COVID-19 outbreak was 28.34%, with a 95% CI of 23.03% to 34.32%. | Discussion |
| Limitations | 25 | Firstly, we excluded studies were not written in English or Chinese. Besides, although subgroup analyses and meta-regression analyses were conducted to control many moderating factors for the pooled prevalence of PTSD symptoms, heterogeneity was still remained in this review. It is reported that heterogeneity is difficult to avoid in meta-analysis of epidemiological surveys, [121] which suggesting the need for caution when drawing inferences about estimates of PTSD in post-disaster research. Additionally, although our study included relevant studies across 30 countries, more than half of the eligible studies were from upper-high income countries. Prevalence studies were scarce for many countries, especially for low-income countries. Considering the inconsistency of the health care environment and socioeconomic status across the world, more prevalence studies in low-income countries are needed to understand the panorama of PTSD among people influenced by COVID-19 outbreak. Also, we noticed that all the included studies were used screening tools to assess PTSD symptoms, no studies included were used diagnostic tools. It is possible that the pooled prevalence of PTSD symptoms caused by COVID-19 outbreak was overestimated in this review. Thus, we think ongoing surveillance is essential. Lastly, some included studies were investigated the prevalence before the time threshold from the first event (usually 30 days), we were unable to check this possible bias between studies. Although we explored the influence of survey time on the pooled prevalence, no significant result was found, which need further clarification. | Discussion |
| Conclusions | 26 | This review has highlighted the importance of considering the psychological impacts of people exposed to the trauma resulting from COVID-19 outbreak. A total of 106713 people exposed to the trauma resulting from the COVID-19 outbreak were identified in the 76 articles, of which 33810 were reported with PTSD symptoms. The pooled prevalence of PTSD symptoms among people exposed to the trauma resulting from COVID-19 outbreak was 28.34%, with a 95% CI of 23.03% to 34.32%. Further research is needed to explore more possible risk factors for PTSD symptoms and identify effective strategies for preventing and treating PTSD symptoms among people exposed to the trauma resulting from COVID-19 outbreak. | Discussion |
| **FUNDING** | | |  |
| Funding | 27 | This research was supported by the Ministry of Science and Technology of China (Grant NO: 2016YFC0900802). | Funding |

*From:*  Moher D, Liberati A, Tetzlaff J, Altman DG, The PRISMA Group (2009). Preferred Reporting Items for Systematic Reviews and Meta-Analyses: The PRISMA Statement. PLoS Med 6(6): e1000097. doi:10.1371/journal.pmed1000097

For more information, visit: **www.prisma-statement.org**.

Page 2 of 2
